# In Vivo Effect of Halicin on Methicillin-Resistant *Staphylococcus aureus*-Infected *Caenorhabditis elegans* and Its Clinical Potential

**DOI:** 10.3390/antibiotics13090906

**Published:** 2024-09-23

**Authors:** Li-Ting Kao, Tsung-Ying Yang, Wei-Chun Hung, Wei-Te Yang, Pu He, Bo-Xuan Chen, Yu-Chi Wang, Shiou-Sheng Chen, Yu-Wei Lai, Hsian-Yu Wang, Sung-Pin Tseng

**Affiliations:** 1 Graduate Institute of Medicine, College of Medicine, Kaohsiung Medical University, Kaohsiung 807, Taiwan; 2 Orthopaedic Research Center, College of Medicine, Kaohsiung Medical University, Kaohsiung 807, Taiwan; 3 Department of Medical Laboratory and Regenerative Medicine, MacKay Medical College, New Taipei 252, Taiwan; 4 Research Institute for Science and Engineering, Waseda University, Tokyo 162-8480, Japan; 5 Department of Microbiology and Immunology, College of Medicine, Kaohsiung Medical University, Kaohsiung 807, Taiwan; 6 School of Chinese Medicine & Graduate Institute of Chinese Medicine, Chinese Medical University, Taichung 404, Taiwan; 7 Department of Medical Laboratory Science and Biotechnology, College of Health Sciences, Kaohsiung Medical University, Kaohsiung 807, Taiwan; 8 Department of Medical Science and Biotechnology, I-Shou University, Kaohsiung 801, Taiwan; 9 Center of General Education, University of Taipei, Taipei 10048, Taiwan; 10 Division of Urology, Taipei City Hospital Renai Branch, Taipei 11221, Taiwan; 11 Graduate Institute of Animal Vaccine Technology, College of Veterinary Medicine, National Pingtung University of Science and Technology, Pingtung 912, Taiwan; 12 Department of Marine Biotechnology and Resources, National Sun Yat-sen University, Kaohsiung 804, Taiwan; 13 Center for Tropical Medicine and Infectious Disease Research, Kaohsiung Medical University, Kaohsiung 807, Taiwan

**Keywords:** methicillin-resistant *Staphylococcus aureus*, halicin, *Caenorhabditis elegans*, in vivo evaluation

## Abstract

Recently, the high proportion of methicillin-resistant *Staphylococcus aureus* infections worldwide has highlighted the urgent need for novel antibiotics to combat this crisis. The recent progress in computational techniques for use in health and medicine, especially artificial intelligence (AI), has created new and potential approaches to combat antibiotic-resistant bacteria, such as repurposing existing drugs, optimizing current agents, and designing novel compounds. Halicin was previously used as a diabetic medication, acting as a c-Jun N-terminal protein kinase (JNK) inhibitor, and has recently demonstrated unexpected antibacterial activity. Although previous efforts have highlighted halicin’s potential as a promising antibiotic, evidence regarding its effectiveness against clinical strains remains limited, with insufficient proof of its clinical applicability. In this study, we sought to investigate the antibacterial activity of halicin against MRSA clinical strains to validate its clinical applicability, and a *C. elegans* model infected by MRSA was employed to evaluate the in vivo effect of halicin against MRSA. Our findings revealed the antibacterial activity of halicin against methicillin-resistant *S. aureus* clinical strains with MICs ranging from 2 to 4 µg/mL. Our study is also the first work to evaluate the in vivo effect of halicin against *S. aureus* using a *C. elegans* model, supporting its further development as an antibiotic.

## 1. Introduction

*Staphylococcus aureus* is a Gram-positive facultative anaerobe [1]. When viewed under a microscope, its resemblance to a cluster of grapes has earned it its name. *S. aureus* is frequently found in different parts of the human body, like the skin, hair, mucous membranes such as the nose and throat, and excrement, typically causing opportunistic illnesses [2]. The infections usually appear as suppurating wounds with different levels of suppurating inflammation and may result in the spread of illnesses such as furuncles, carbuncles, otitis media, sinusitis, osteomyelitis, bloodstream infection, and sepsis [3]. The treatment of such infections frequently involves beta-lactams like methicillin, macrolides, aminoglycosides, and quinolones [4]. Despite this, the rise of methicillin-resistant *S. aureus* (MRSA) was first documented in 1961 [5]. The methicillin resistance is attributed to the acquisition of the *mecA* gene, encoding PBP2a through mobile genetic elements [6]. Studies have demonstrated that the PBP2a protein is mainly responsible for making most β-lactam drugs ineffective [7], thereby causing drug resistance. Thus, the methicillin resistance can also extend to a wide range of β-lactam drugs, rendering the treatment ineffective [8]. 

Endemic MRSA outbreaks began in 1999 and developed into a global problem [9], and the spread of MRSA has become increasingly serious due to the difficulty of infection control [10,11]. Currently, it is estimated that more than 53 million people worldwide are MRSA carriers [12], causing infections in hospitals and excess medical costs of USD 27,000–34,000 [13]. According to a previous review [14], the prevalence of MRSA across the world ranged from 14 to 74%: the percentage of methicillin resistance in Europe (except Germany) was about 40%, in Brazil in South America it was about 50%, and reports from the United States estimated between 30 and 50%. A study from Nepal in South Asia reported that among 1804 samples from patients with *S. aureus* infection in 2021, 1027 of them were MRSA (1027/1804, 57%) [15]. Earlier, in Taiwan, the proportion of nosocomial infections caused by MRSA (hospital-acquired MRSA, also known as healthcare-associated MRSA) increased annually from 75% in 1998 to 84% in 2000 [16]. In a study conducted in 2016 in Taiwan [17], 177 of 307 *S. aureus* isolated from skin and soft tissue infections were MRSA (177/307, 57.7%), among which 40% of the patients suffered from a community-associated MRSA infection, highlighting that there is an MRSA issue within the Taiwanese community. The previous study focusing on community-associated MRSA in Taiwan revealed that 3% of the population in the community were MRSA carriers, helping to spread MRSA [18]. In addition to the high rate of nosocomial infections, the spread of MRSA in the community is also an important issue that contributes to methicillin and other antibiotic resistance, thereby highlighting the urgent need for the development of novel agents against MRSA.

The recent progress in computational techniques for use in health and medicine [19], especially artificial intelligence (AI), has created new and potential approaches to combat antibiotic-resistant bacteria, such as repurposing existing drugs, optimizing current agents, and designing novel compounds [20]. The utilization of AI in the identification of innovative antimicrobial compounds from drug databases has become a prevailing trend [21], exhibiting significant advancements. Stokes et al. pinpointed “halicin” (initially known as SU3327) from an extensive collection of chemical compounds through machine learning techniques [22]. Halicin was previously used as a diabetic medication, acting as a c-Jun N-terminal protein kinase (JNK) inhibitor. Recent studies, including that by Stokes et al., revealed unexpected antibacterial activities for halicin [22,23,24,25,26,27,28,29,30]. The remarkable antimicrobial effects of halicin were revealed against Enterobacterales, *S. aureus*, *Mycobacterium tuberculosis*, and *Clostridium* species. Furthermore, halicin has demonstrated successful therapeutic results in the treatment of intestinal infections in mice caused by *Clostridium difficile* and skin infections provoked by pan-resistant *A. baumannii* [22]. Halicin also possesses strong anti-biofilm effects against *S. aureus*. Additionally, studies suggest that halicin interferes with the electrical gradient of bacterial membranes and increases the expression of bacterial genes associated with iron homeostasis, resulting in disturbances to pH regulation across the bacterial cell membrane and ultimately inhibiting bacterial growth [22,31]. As a result, the bacteria might not acquire resistance to this novel mode of action.

Encouraged by previous efforts, halicin could be a promising agent to combat MRSA. However, the evidence of halicin’s effects on clinical strains remains limited, lacking proof of its clinical applicability. In this study, we sought to investigate the antibacterial activity of halicin against MRSA clinical strains to validate its clinical applicability, and a *C. elegans* model infected by MRSA was employed to evaluate the in vivo effect of halicin against MRSA.

## 2. Results

### 2.1. Characterization of MRSA Strains in This Study

**Figure 1:**
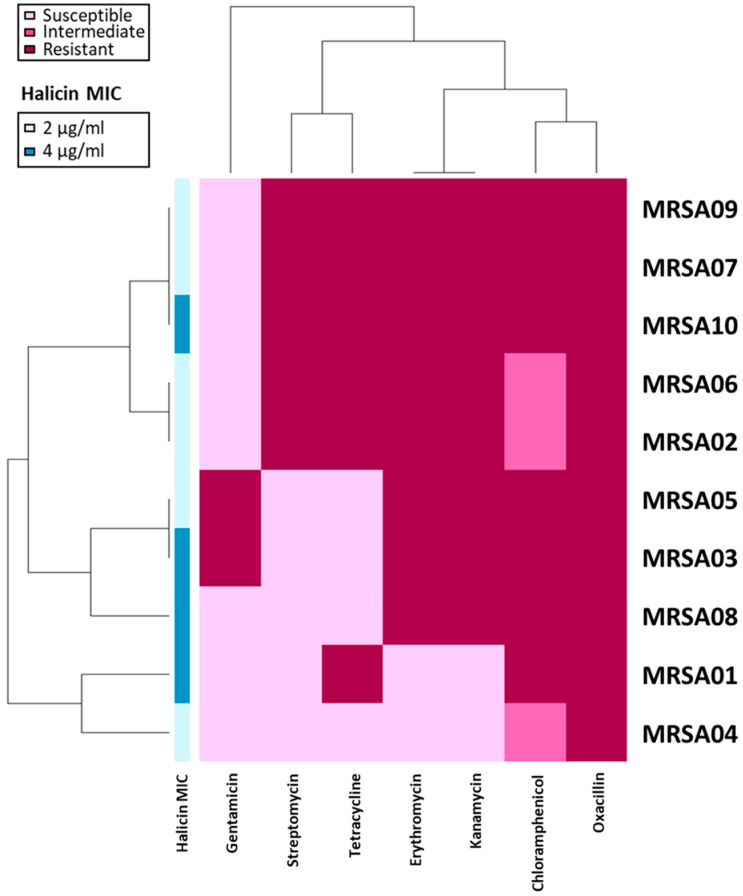
Heatmap of antimicrobial-resistant profiles in 10 MRSA isolates. Antibiotic susceptibilities: light pink, susceptible; pink, intermediate; dark pink, resistant. Differences between isolate groups were categorized according to antibiotic-resistant profiles. The MICs of halicin were transformed as annotations along the left side of the heatmap.

The results of antimicrobial susceptibility testing are shown in Figure 1. Among the 10 methicillin-resistant *S. aureus* (MRSA) strains tested in this study, of 80% were resistant to erythromycin and kanamycin, and 60% of the strains were resistant to tetracycline. All the strains were non-susceptible to chloramphenicol, with 70% being resistant and 30% being intermediate-resistant. A relatively remarkable susceptibility was found for gentamicin, which showed a resistant rate of only 20%. However, the antimicrobial susceptibility testing highlights the urgent need for novel antimicrobials against MRSA.

**Figure 2:**
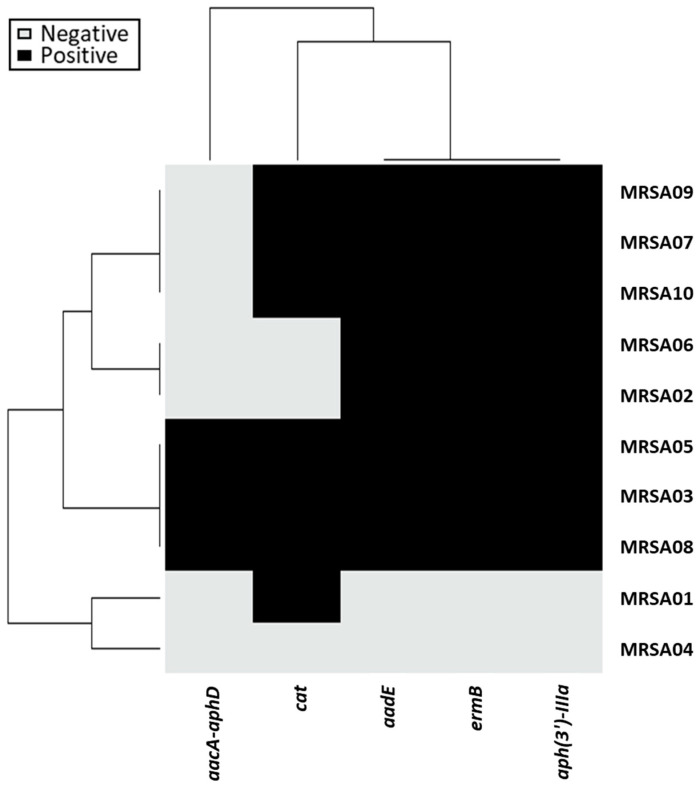
Heatmap of the antibiotic-resistant genes in 10 MRSA isolates. Black and gray colors indicate positive and negative PCR detection results, respectively. Differences between isolate clusters were categorized according to gene carriages or distributions.

The PCR detection of mobile antibiotic-resistant determinants is shown in Figure 2. Of the five genes we detected, the *ermB* gene, which confers erythromycin resistance, was most common (80%). The *aph(3′)-IIIa*, *aadE*, and *aacA-aphD* genes, which correspond to aminoglycoside resistance, were found in 80%, 80%, and 30% of the strains, respectively. The *cat* gene was observed in 70% of the strains that showed resistant phenotypes in the antimicrobial susceptibility testing. Notably, three strains were harbored by all five genes we detected, implying the potential for the horizontal transfer of these antibiotic-resistant determinants.

The distribution of virulence factors is listed in Table 1. The *seb*, *hlb*, and *scn* genes were detected in all of the strains (100%), followed by the PVL gene in 80% of the strains. Both the *sak* and *sep* genes were found in 30% of the strains. None of the MRSA strains we detected were found to contain the *sea* gene. The results of the virulence factor detection revealed that the clinical strains in this study were highly virulent.

### 2.2. Antibacterial Activity of Halicin

Halicin’s antibacterial activity against the five reference strains and ten clinical strains was investigated in vitro. As shown in Table 2, the MIC values of halicin against MSSA ATCC 29213, MRSA ATCC 33592, MRSA USA300, hVISA Mu3, and VISA Mu50 were 2, 2, 4, 2, and 1 µg/mL, respectively. The MIC results against the MRSA clinical strains are demonstrated in Figure 1 and Table 3. Among the ten strains we examined, the MICs were revealed as 2 µg/mL for six strains and 4 µg/mL for the remaining four strains, with the MIC_50_ and MIC_90_ of 2 and 4 µg/mL, respectively. Compared to the antimicrobial susceptibility profiles of the 10 isolates, no correlation was found between the MICs of halicin and the resistance to the clinical antibiotics. Our findings supported that the antibacterial activity of halicin against the MRSA clinical strains was steady and not affected by resistance to the clinical antibiotics, implying the potential for halicin to be used as an anti-MRSA agent.

### 2.3. In Vivo Assessment of Halicin

**Figure 3:**
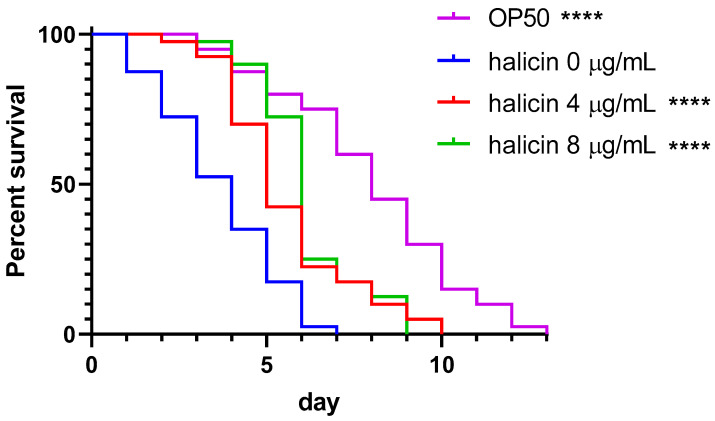
Survival curve of nematodes infected by methicillin-resistant *S. aureus* USA300 and treated with 0, 4, or 8 µg/mL of halicin. ****, *p* < 0.0001 compared to the group of OP50.

To investigate the in vivo efficacy of halicin against MRSA, *C. elegans* animals infected with MRSA USA300 were employed. Compared to the untreated control group, halicin resulted in a significant right-shift in the survival curve against the MRSA USA300 in the 1× and 2× MIC (both *p* < 0.0001) treatment groups (Figure 3). The median nematode survival times were significantly extended to 5.5 days (*p* < 0.0001) at 1× MIC and 6 days (*p* < 0.0001) at 2× MIC (Table 4), with significant decreases in mortality risk observed at both 1× MIC [hazard ratio (HR) 0.493; 95% confident interval (CI) 0.309 to 0.785] and 2× MIC (HR 0.432; 95% CI 0.267 to 0.698). Similar findings were observed for those infected with MRSA03, which possessed the most virulent genes. The significantly increased survival indicated the in vivo activity against the clinical MRSA strain (Supplementary Figure S1). The remarkable in vivo effects of halicin on MRSA-infected *C. elegans* further proved its potential application in clinical settings.

## 3. Discussion

The high rate and spread of MRSA infections in the community represents an important issue that contributes to resistance to methicillin and other antibiotics, thereby highlighting the urgent need for the development of novel agents against MRSA. Given the attractiveness of using computational approaches as a strategy for this issue, Stokes et al., using a deep learning approach, revealed that halicin (i.e., SU3327), which is a potent, selective, and substrate-competitive JNK inhibitor [32], represented a broad-spectrum bactericidal antibiotic [22]. In their study, halicin showed remarkable antibacterial activities against *Escherichia coli* lab strain BW25113 carrying various antibiotic-resistant genes (*mcr-1*, *cat*, *bla*_OXA-1_, *aac(6′)-Ib-cr*, and so on), with MICs of 0.5–2 µg/mL. Furthermore, the results against a panel of carbapenem-resistant Enterobacterales demonstrated a similar result, with a MICs ranging between 1 and 16 µg/mL [22]. Although the antibacterial activities against multidrug-resistant *Acinetobacter baumannii* were also remarkable, those against multidrug-resistant *Pseudomonas aeruginosa* were far from ideal. In their study, a further mouse wound model infected with *A. baumannii* was used which revealed a significant positive treatment effect [22]. Notably, the antibacterial spectrum of halicin also involved *Mycobacterium tuberculosis* and *Clostridium difficile*, where the total eradication of *C. difficile* by halicin was noticed in an in vivo mouse model in 5 days [22]. However, the antibacterial activity of halicin against *S. aureus* was not mentioned in their study. 

The current work examined the clinical applicability of halicin using a batch of clinical methicillin-resistant *S. aureus* strains, with a MIC range of 2–4 µg/mL. The results did not correlate to the antimicrobial susceptibility profiles (Figure 1). The antibacterial mechanism of halicin was to disrupt the membrane potential and lead to bacterial death, which is quite different from traditional antibiotics and can bypass the resistance. The cut-off value between susceptibility and resistance will need further investigation on pharmacokinetics and pharmacodynamics. However, the MICs of halicin (2–4 µg/mL)) were below or equal to the most resistant breakpoints of the antibiotics tested in this study, including oxacillin (4 µg/mL), gentamicin (16 µg/mL), tetracycline (16 µg/mL), erythromycin (8 µg/mL), and chloramphenicol (32 µg/mL). The results of the *C. elegans* model revealed the potential in vivo activity, with a prolonged medium survival time from 4 to 6 days. Our findings agreed with some of the previous studies.

Recently, halicin has attracted researchers’ attention to evaluate its anti-*S. aureus* effect [23,24,25,26,27,28,29,30]. In an earlier study, Booq et al. reported a different perspective on halicin [30], with a comparison between freshly prepared and older halicin solutions. However, high MICs were revealed for *S. aureus* ATCC BAA-977 (16 µg/mL) even using a freshly prepared halicin solution. The MICs of older halicin solutions against those strains were 32 µg/mL, with no notable difference found. The findings by Booq et al. disagreed with the previous findings [22], implying the need for further investigation. In the previous study conducted in China, Li et al. investigated the antibacterial effect of halicin against *S. aureus*, with MICs ranging from 2 to 4 µg/mL [29]. The biofilm inhibition was also first revealed in their study, with a nearly 50% reduction of biofilm mass discovered compared to untreated control (*p* < 0.05). In another study, Gent et al. reported the synergism and antibiofilm activity of halicin and the synthetic antibacterial and antibiofilm peptide (SAAP-148) against *E. coli* and *S. aureus* [28]. The 3-log reduction in the bacterial load of the *S. aureus* biofilms adhered to silicone disks was noticed after 4 h exposure to the combinations of SAAP-148 and halicin (*p* < 0.0001). Additionally, a 3D human epidermal model was employed to investigate the treatment effect of halicin and its combinations with other drugs against *S. aureus* [28]. After 4 h of exposure to 102.4 or 204.8 µM (ca. 26.75 or 53.5 µg/mL) of halicin, only one to two logs of bacterial reduction were found, illustrating that the single regimen of halicin could not treat the infection model; on the other hand, the effects of the single regimen of SAAP-148 demonstrated a nearly 2-log reduction in this model. The combined therapy of halicin and SAAP-148 revealed significant eradication at 12.8 µM of SAAP-148 plus 102.4 µM of halicin compared to their respective single regimens (*p* < 0.001) [28]. Their ex vivo models first provided insight into the in vivo application of halicin, yet the concentration they used for halicin and its efficacy needs to be further confirmed. Furthermore, the combination of SAAP-148 and halicin was revealed to be a novel agent combating the Gram-negative bacteria-colonizing catheters [27].

In the study also focusing on the antibiofilm activity, Higashihira et al. described the anti-biofilm activities of halicin against *S. aureus* strain Xen-36 [26], of which the MIC to halicin was 25 µM (ca. 6.5 µg/mL). In their biofilm inhibition assays, the biofilms were left to stand for three or seven days and then treated with halicin at various concentrations, with the biomass and viable count in the biofilm being determined. Against the 3-day-old biofilm, halicin significantly deformed >75% of the biofilm at concentrations higher than 25 µM (*p* < 0.001) [26], with nearly a 70% reduction found for that treated with 12.5 µM of halicin. Similarly, >75% reduction of viable cells was observed at concentrations higher than 25 µM (*p* = 0.004), and nearly 75% reduction was found for that treated with 12.5 µM of halicin. Although the biomass of the seven-day biofilms seemed to be hard to eradicate, halicin still significantly reduced the biomass at 100 µM (*p* = 0.04) and 200 µM (*p* = 0.002) [26]. Despite the biomass of the biofilm remaining constant, the viable counts in the biofilm were significantly reduced, with nearly 50%, 70%, >75%, and >75% reductions found at 25, 50, 100, and 200 µM, respectively. These findings agreed with those of Li et al. in 2021 [29]. Encouraged by the previous study [26], in another study conducted by Higashihira et al., the ex vivo antibiofilm study was performed using several orthopedically relevant substrates [25], such as titanium alloy, cobalt-chrome, ultra-high molecular weight polyethylene, devitalized muscle, or devitalized bone. For the less-mature biofilm (24 h) with *S. aureus* Xen-36, the antibiotic penetration of halicin and vancomycin were comparable to that in the devitalized muscle model, with 40× the MIC of halicin or the 50× MIC of vancomycin being required to eradicate *S. aureus* Xen-36 [25]. Despite vancomycin showing acceptable activity in the devitalized muscle with a less-mature biofilm, the necessary concentration of vancomycin to treat the devitalized bone with the less-mature biofilm was 100× MIC, significantly higher than that of halicin (also 40× MIC). In both the devitalized muscle and bone models with mature biofilms (7 days), halicin continued to exert more significant activities against biofilms than vancomycin (*p* < 0.01) [25]. The findings suggested that halicin is a promising agent for animal models of orthopedic infection.

In the previous study conducted in China, Wang et al. comprehensively evaluated the potential of halicin as a veterinary agent against *S. aureus* [24], with an MIC and a minimum bactericidal concentration (MBCs) of 8 and 16 µg/mL, respectively. The post-antibiotic effects (PAE) were noticed for halicin against *S. aureus* ATCC 29213 for 1.45 h [24]. Notably, even at concentrations far lower than the MICs, halicin still demonstrated the post-antibiotic sub-minimum inhibitory concentration effects (PASME) against *S. aureus* ATCC 29213 for 1.89 to 3.24 h, where the bacterial growth was postponed. Their time-killing assays illustrated a bactericidal activity for halicin against *S. aureus* ATCC 29213 [24], with an inhibitory effect observed for the 1/2× MIC at 24 h. Additionally, nearly no evolution of resistant mutation was noticed for *S. aureus* ATCC 29213, even after a 40-day co-culture, suggesting the steady antibacterial activity of halicin against *S. aureus*. In further investigation, Zhang et al. evaluated halicin’s safety as well as its efficacy [23]. In their study, the MICs against *S. aureus* strains were all 8 µg/mL, which agreed with the results in the study by Stock et al. [22]. Additionally, toxicological results similar to the previous study found that the LD_50_ of halicin in the oral route in mice was 2018.3 mg/kg [24], and no obvious genotoxicity was found. However, with 90-day oral administration, sub-chronic toxicity was observed at a high dose of 201.8 mg/kg, accompanied by weight loss and slight renal inflammation. The studies supported the safety and potential of using halicin to treat infections.

## 4. Materials and Methods

### 4.1. Bacterial Collection

A total of 10 methicillin-resistant *S. aureus* (MRSA) were obtained from the previous study [33]. The reference strains, including methicillin-susceptible *S. aureus* ATCC 29213, MRSA ATCC 33592, MRSA USA300, heterogeneous vancomycin-intermediate *S. aureus* (hVISA) Mu3, and VISA Mu50 were also employed for analyses in this study. All cultures were stored at −80 °C with the addition of 20% glycerol. The culture was recovered and subcultured once before the experiments.

### 4.2. Antimicrobial Susceptibility Testing

The antimicrobial susceptibility testing was conducted using the standard agar dilution methods in accordance with the guidelines of the Clinical & Laboratory Standards Institute (CLSI) for tetracycline, erythromycin, kanamycin, gentamicin, streptomycin, and chloramphenicol [34]. Quality control between batches was performed using *S. aureus* ATCC 29213 reference strain.

For the antibacterial activity of halicin, the strains were examined using the broth microdilution method according to the CLSI guidelines [34]. Briefly, halicin (Development Center for Biotechnology, Taiwan) was dissolved in water and diluted with cation-adjusted Mueller-Hinton broth (CAMHB) (BD Difco, Sparks, MD, USA) to 0.5–32 µg/mL in the final concentration. The *S. aureus*, cultured overnight on a blood agar plate (BD Difco, Sparks, MD, USA), was suspended in CAMHB and inoculated into wells at 5 × 10^5^ colony-forming units per mL (CFU/mL). The plate was incubated at 37 °C for 24 h. The microplate reader was employed to determine the bacterial growth via the change in the absorbance. The minimum inhibitory concentration of halicin was defined as the lowest concentration with no viable growth. Every experiment was repeated at least three times.

### 4.3. Gene Detection

The polymerase chain reaction (PCR) was applied to detect the presence of the following antibiotic-resistant genes: aminoglycoside (*aph(3′)-IIIa*, *aadE*, and *aacA-aphD*), erythromycin (*ermB*), and chloramphenicol (*cat*) using primers in the previous study [35]. Virulence genes, including Panton-Valentine Leukocidin (PVL), staphylococcal enterotoxins A, B, and P (*sea*, *seb*, and *sep*), beta hemolysin (*hlb*), chemotaxis inhibitory protein (*chp*), staphylokinase (*sak*), and staphylococcal complement inhibitor (*scn*), were also detected according to the previous study [36]. All detections were performed with respective positive controls, and sequencing was conducted to validate those positive results.

### 4.4. Caenorhabditis Elegans In Vivo Study

The N2 strain of *Caenorhabditis elegans* is utilized in this research. The worms are fed with *E. coli* OP50 bacterial lawns as the nutrient source on nematode growth medium (NGM) at 20 °C. All experimental procedures were executed as described in the previous study [37]. Briefly, the NGM plates with over 80% of mature worms were washed with sterilized ddH_2_O, and the nematodes and eggs were transferred to a 15 mL centrifuge tube. After washing thrice with ddH_2_O to remove most of the residual *E. coli* OP50, the supernatant was removed after centrifugation with 3 mL of reserved supernatant. Subsequently, 0.5 mL of 5 M potassium hydroxide (KOH) and 1 mL of sodium hypochlorite (NaOCl) were added to break down the nematodes’ bodies and keep their eggs. The neutralization was conducted by adding 10 mL of ddH_2_O. After neutralization, the eggs were collected and washed thrice with ddH_2_O to eliminate alkaline influence. The eggs were then resuspended in 1 mL of M9 buffer and placed in an empty 3.5 mm Petri dish. The eggs were incubated at 20 °C for 24 h to allow them to hatch and reach the L1 stage. The L1 larvae were then transferred onto NGM agar with *E. coli* OP50 bacterial lawns, with 2500–3000 worms per plate cultured at 20 °C for 44 h until synchronously reaching the L4 stage for further experiments.

The agar plates with halicin at 0×, 1×, and 2× MIC were prepared and spread with MRSA USA300. The plates were incubated at room temperature overnight to allow bacterial growth. 40 growth-synchronized L4-stage nematodes were placed onto the plates with bacterial lawns of MRSA USA300. Daily observations and counting of worms were conducted, with nematode plates replaced every two days to mitigate the impact of maternal egg-laying and worms hatching on the results. When transferring onto new plates, the nematodes were observed for one hour post-operation to ensure no artificial error. Nematode-containing plates were held at 25 °C, with nematode survival recorded daily. Worms that died from crawling off the plate or transferring were censored from the analysis. All assays were performed in triplicate biologically.

### 4.5. Statistical Analysis

Visualization of antibiotic susceptibility and gene detection profiles were achieved through the generation of heatmaps using ggplot2 in RStudio (v. 1.1.453). Kaplan–Meier curves were generated for survival tests utilizing GraphPad Prism software (v. 8.0) and subsequently assessed through Mantel–Haenszel Cox log-rank tests.

## 5. Conclusions

In this study, we revealed the antibacterial activity of halicin against methicillin-resistant *S. aureus* clinical strains, with MICs ranging from 2 to 4 µg/mL, agreeing with most of the previous findings [23,24,25,26,27,28,29]. Our study was the first work to evaluate the in vivo effect of halicin against *S. aureus* using a *C. elegans* model, in which only an innate immunity is presented [38], excluding the interference of the adaptive immunity during the in vivo assessment. The significant extension of the nematodes’ median survival time suggested the remarkable treatment effect of halicin against *S. aureus*, supporting its further development as an antibiotic.

## Figures and Tables

**Table 1:** Distribution of virulence factors in the 10 MRSA clinical strains tested in this study.

Clinical Isolate	PVL	*seb*	*hlb*	*chp*	*sak*	*scn*	*sea*	*sep*
MRSA01	+	+	+	+	−	+	−	−
MRSA02	+	+	+	+	−	+	−	−
MRSA03	+	+	+	−	+	+	−	+
MRSA04	+	+	+	+	−	+	−	−
MRSA05	−	+	+	−	+	+	−	+
MRSA06	+	+	+	+	−	+	−	−
MRSA07	+	+	+	+	−	+	−	−
MRSA08	−	+	+	−	+	+	−	+
MRSA09	+	+	+	+	−	+	−	−
MRSA10	+	+	+	+	−	+	−	−
Total proportion	80%	100%	100%	70%	30%	100%	0%	30%

**Table 2:** MICs of halicin against five *S. aureus* reference strains.

Lab Strain ^1^	Halicin MIC (µg/mL)
MSSA ATCC 29213	2
MRSA ATCC 33592	2
MRSA USA300	4
hVISA Mu3	2
VISA Mu50	1

^1^ MSSA, methicillin-susceptible *S. aureus*; MRSA, methicillin-resistant *S. aureus*; hVISA, heterogeneous vancomycin-intermediate *S. aureus*; VISA, vancomycin-intermediate *S. aureus*.

**Table 3:** MIC distribution of halicin against 10 MRSA clinical strains we examined.

**Methicillin-Resistant** ***S. aureus* Clinical Strains**	**Halicin**			
	**MIC Range (µg/mL)**	**MIC_50_ (µg/mL)**	**MIC_90_ (µg/mL)**	**Mode of MIC (µg/mL)**
	2–4	2	4	2

**Table 4:** Statistical analysis and summary of the nematode survival.

Treatment	Median Time (Days)	*p* Value	Hazard Ratio	95% Confidence Interval (CI)	
				Lower	Upper
control	4	-	Reference		
4 µg/mL halicin	5.5	<0.0001	0.493	0.309	0.785
8 µg/mL halicin	6	<0.0001	0.432	0.267	0.698

## Data Availability

The original contributions presented in the study are included in the article/Supplementary Materials, further inquiries can be directed to the corresponding authors.

## Supplementary Materials

The following supporting information can be downloaded at: https://www.mdpi.com/article/10.3390/antibiotics13090906/s1, Figure S1: Survival curve of nematodes infected by methicillin-resistant *S. aureus* clinical strain. MRSA03 and treated with 0, 4, or 8 μg/mL of halicin. ****, *p* < 0.0001 compared to the group of OP50.

## References

[1] Foster T.J. (2002). *Staphylococcus* *aureus*. Mol. Med. Microbiol..

[2] Howden B.P., Giulieri S.G., Wong Fok Lung T., Baines S.L., Sharkey L.K., Lee J.Y.H., Hachani A., Monk I.R., Stinear T.P. (2023). *Staphylococcus aureus* host interactions and adaptation. Nat. Rev. Microbiol..

[3] Taylor T.A., Unakal C.G. (2024). Staphylococcus aureus Infection. StatPearls.

[4] David M.Z., Daum R.S. (2017). Treatment of *Staphylococcus aureus* infections. Curr. Top Microbiol. Immunol..

[5] Barber M. (1961). Methicillin-resistant staphylococci. J. Clin. Pathol..

[6] Stapleton P.D., Taylor P.W. (2002). Methicillin resistance in *Staphylococcus aureus*: Mechanisms and modulation. Sci. Prog..

[7] Peacock S.J., Paterson G.K. (2015). Mechanisms of methicillin resistance in *Staphylococcus aureus*. Annu. Rev. Biochem..

[8] Bæk K.T., Gründling A., Mogensen R.G., Thøgersen L., Petersen A., Paulander W., Frees D. (2014). β-Lactam resistance in methicillin-resistant *Staphylococcus aureus* USA300 is increased by inactivation of the ClpXP protease. Antimicrob. Agents Chemother..

[9] Hryniewicz W. (1999). Epidemiology of MRSA. Infection.

[10] Herwaldt L.A. (1999). Control of methicillin-resistant *Staphylococcus aureus* in the hospital setting. Am. J. Med..

[11] Pittet D., Hugonnet S., Harbarth S., Mourouga P., Sauvan V., Touveneau S., Perneger T.V. (2000). Effectiveness of a hospital-wide programme to improve compliance with hand hygiene. Infection Control Programme. Lancet.

[12] Okuma K., Iwakawa K., Turnidge J.D., Grubb W.B., Bell J.M., O’Brien F.G., Coombs G.W., Pearman J.W., Tenover F.C., Kapi M. (2002). Dissemination of new methicillin-resistant *Staphylococcus aureus* clones in the community. J. Clin. Microbiol..

[13] Abramson M.A., Sexton D.J. (1999). Nosocomial methicillin-resistant and methicillin-susceptible *Staphylococcus aureus* primary bacteremia: At what costs?. Infect. Control Hosp. Epidemiol..

[14] Köck R., Becker K., Cookson B., van Gemert-Pijnen J., Harbarth S., Kluytmans J., Mielke M., Peters G., Skov R., Struelens M. (2010). Methicillin-resistant *Staphylococcus aureus* (MRSA): Burden of disease and control challenges in Europe. Euro. Surveill..

[15] Pradhan P., Rajbhandari P., Nagaraja S., Shrestha P., Grigoryan R., Satyanarayana S., Davtyan H. (2021). Prevalence of methicillin-resistant *Staphylococcus aureus* in a tertiary hospital in Nepal. Public Health Action.

[16] Hsueh P.R., Liu C.Y., Luh K.T. (2002). Current status of antimicrobial resistance in Taiwan. Emerg. Infect. Dis..

[17] Changchien C.-H., Chen S.-W., Chen Y.-Y., Chu C. (2016). Antibiotic susceptibility and genomic variations in *Staphylococcus aureus* associated with skin and soft tissue infection (SSTI) disease groups. BMC Infect. Dis..

[18] Lu P.-L., Chin L.-C., Peng C.-F., Chiang Y.-H., Chen T.-P., Ma L., Siu L. (2005). Risk factors and molecular analysis of community methicillin-resistant *Staphylococcus aureus* carriage. J. Clin. Microbiol..

[19] Rajpurkar P., Chen E., Banerjee O., Topol E.J. (2022). AI in health and medicine. Nat. Med..

[20] Branda F., Scarpa F. (2024). Implications of artificial intelligence in addressing antimicrobial resistance: Innovations, global Challenges, and healthcare’s future. Antibiotics.

[21] Masoudi-Sobhanzadeh Y., Omidi Y., Amanlou M., Masoudi-Nejad A. (2020). Drug databases and their contributions to drug repurposing. Genomics.

[22] Stokes J.M., Yang K., Swanson K., Jin W., Cubillos-Ruiz A., Donghia N.M., MacNair C.R., French S., Carfrae L.A., Bloom-Ackermann Z. (2020). A deep learning approach to antibiotic discovery. Cell.

[23] Zhang M., Lin S., Han L., Zhang J., Liu S., Yang X., Wang R., Yang X., Yi Y. (2024). Safety and efficacy evaluation of halicin as an effective drug for inhibiting intestinal infections. Front. Pharmacol..

[24] Wang S., Zhao K., Chen Z., Liu D., Tang S., Sun C., Chen H., Wang Y., Wu C. (2024). Halicin: A new horizon in antibacterial therapy against veterinary pathogens. Antibiotics.

[25] Higashihira S., Simpson S.J., Morita A., Suryavanshi J.R., Arnold C.J., Natoli R.M., Greenfield E.M. (2024). Halicin remains active against *Staphylococcus aureus* in biofilms grown on orthopaedically relevant substrates. Bone Jt. Res..

[26] Higashihira S., Simpson S.J., Collier C.D., Natoli R.M., Kittaka M., Greenfield E.M. (2022). Halicin is effective against *Staphylococcus aureus* biofilms in vitro. Clin. Orthop. Relat. Res..

[27] Bouhrour N., van der Reijden T.J.K., Voet M.M., Schonkeren-Ravensbergen B., Cordfunke R.A., Drijfhout J.W., Bendali F., Nibbering P.H. (2023). Novel antibacterial agents SAAP-148 and halicin combat Gram-negative bacteria colonizing catheters. Antibiotics.

[28] van Gent M.E., van der Reijden T.J.K., Lennard P.R., de Visser A.W., Schonkeren-Ravensbergen B., Dolezal N., Cordfunke R.A., Drijfhout J.W., Nibbering P.H. (2022). Synergism between the synthetic antibacterial and antibiofilm peptide (SAAP)-148 and halicin. Antibiotics.

[29] Li H., Xu L., Liu Y., She P., Wu Y. (2021). Antibacterial effects of small molecule antidiabetic agent halicin against *Staphylococcus aureus*. Chin. J. Lab. Med..

[30] Booq R.Y., Tawfik E.A., Alfassam H.A., Alfahad A.J., Alyamani E.J. (2021). Assessment of the Antibacterial Efficacy of Halicin against Pathogenic Bacteria. Antibiotics.

[31] Jang S., Javadov S. (2014). Inhibition of JNK aggravates the recovery of rat hearts after global ischemia: The role of mitochondrial JNK. PLoS ONE.

[32] Gehringer M., Muth F., Koch P., Laufer S.A. (2015). c-Jun N-terminal kinase inhibitors: A patent review (2010–2014). Expert Opin. Ther. Pat.

[33] Hung W.C., Wan T.W., Kuo Y.C., Yamamoto T., Tsai J.C., Lin Y.T., Hsueh P.R., Teng L.J. (2016). Molecular evolutionary pathways toward two successful community-associated but multidrug-resistant ST59 methicillin-resistant *Staphylococcus aureus* Lineages in Taiwan: Dynamic modes of mobile genetic element salvages. PLoS ONE.

[34] Clinical & Laboratory Standards Institute (CLSI) (2024). Performance Standards for Antimicrobial Susceptibility Testing: Twenty-Seventh Informational Supplement. Document M100-S34 CLSI.

[35] van Wamel W.J., Rooijakkers S.H., Ruyken M., van Kessel K.P., van Strijp J.A. (2006). The innate immune modulators staphylococcal complement inhibitor and chemotaxis inhibitory protein of *Staphylococcus aureus* are located on beta-hemolysin-converting bacteriophages. J. Bacteriol..

[36] Takano T., Higuchi W., Zaraket H., Otsuka T., Baranovich T., Enany S., Saito K., Isobe H., Dohmae S., Ozaki K. (2008). Novel characteristics of community-acquired methicillin-resistant *Staphylococcus aureus* strains belonging to multilocus sequence type 59 in Taiwan. Antimicrob. Agents Chemother..

[37] Chang H.C., Huang Y.T., Chen C.S., Chen Y.W., Huang Y.T., Su J.C., Teng L.J., Shiau C.W., Chiu H.C. (2016). In vitro and in vivo activity of a novel sorafenib derivative SC5005 against MRSA. J. Antimicrob. Chemother..

[38] Ermolaeva M.A., Schumacher B. (2014). Insights from the worm: The *C. elegans* model for innate immunity. Semin. Immunol..

