# Supplementary material for: In Vivo Effect of Halicin on Methicillin-Resistant Staphylococcus aureus-Infected Caenorhabditis elegans and Its Clinical Potential
Source: Antibiotics (Basel). 2024 Sep 23;13(9):906. doi: 10.3390/antibiotics13090906 (PMC11429483; doi:10.3390/antibiotics13090906)
Supplement: Supplementary file 1 [file antibiotics-13-00906-s001.zip › antibiotics-3167468-supplementary.pdf]

antibiotics

MDPI

Supplementary Materials

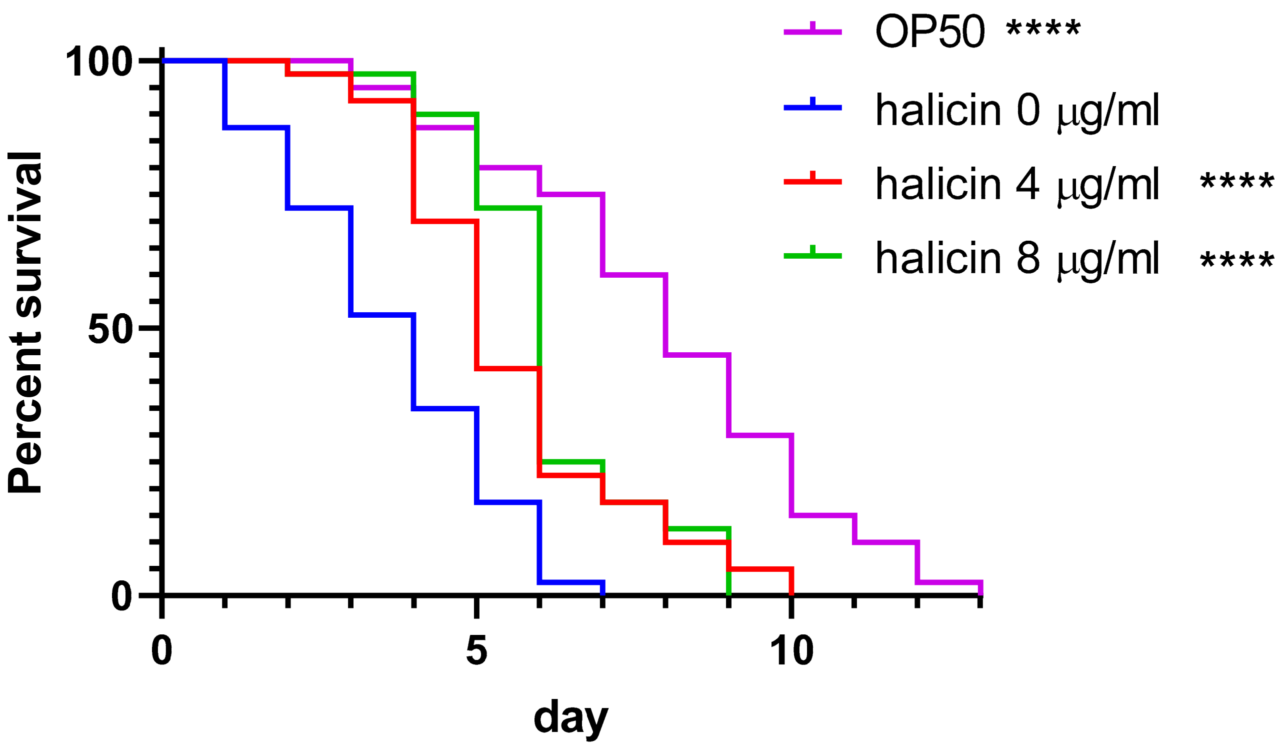

Figure S1. Survival curve of nematodes infected by methicillin-resistant *S. aureus* clinical strain MRSA03 and treated with 0, 4, or 8 µg/ml of halicin. ****, $p<0.0001$ compared to the group of OP50.
